# Investigation of the impact of copper nanoparticles coated with ocimum bassilicum at chemoradiotherapy of colon carcinoma

**DOI:** 10.1016/j.bbrep.2024.101780

**Published:** 2024-07-06

**Authors:** Farshad Seyed Nejad, Mostafa Alizade-Harakiyan, Mehdi Haghi, Rokhsareh Ebrahimi, Mohammad Mahdi Zangeneh, Alireza Farajollahi, Roghayeh Fathi, Reza Mohammadi, Samira Samadi Miandoab, Mohammad Heydarnezhad Asl, Baharak Divband, Amin Ahmadi

**Affiliations:** a Molecular Medicine Research Center, Tabriz University of Medical Sciences, Tabriz, Iran; b Department of Radiation Oncology, Faculty of Medicine, Tabriz University of Medical Sciences, Tabriz, Iran; c Medical Physics Department, Faculty of Medicine, Tabriz University of Medical Sciences, Tabriz, Iran; d Department of Animal Biology, Faculty of Natural Sciences, University of Tabriz, Tabriz, Iran; e Medicinal Chemistry Department, Faculty of Pharmacy, Tabriz University of Medical Sciences, Tabriz, Iran; f Biotechnology and Medicinal Plants Research Center, Ilam University of Medical Sciences, Ilam, Iran; g Medical Radiation Science Research Team, Tabriz University of Medical Sciences, Tabriz, Iran; h Polymer Research Laboratory, Department of Organic and Biochemistry, University of Tabriz, Tabriz, Iran; i Department of Inorganic Chemistry, Faculty of Chemistry, University of Tabriz, Tabriz, Iran; j Research Center for Pharmaceutical Nanotechnology, Biomedicine Institute, Tabriz University of Medical Sciences, Tabriz, Iran

**Keywords:** Copper nanoparticles, *Ocimum basillicum* extract, Colon cancer, Radioteraphy

## Abstract

**Background:**

Colon carcinoma poses a significant health challenge globally, particularly in developed nations where sedentary lifestyles, poor dietary choices, and genetic factors play a crucial role in its prevalence. Chemotherapy, the primary treatment method, carries severe side effects that can jeopardize patients' lives. Herbal extracts such as Ocimum Basillicum extract have shown effectiveness against cancer cells. Additionally, nanoparticles can significantly enhance drug delivery efficacy in these scenarios.

**Aim:**

This article aims to investigate the impact of copper nanoparticles coated with Ocimum Bassilicum at chemoradiotherapy of Colon Carcinoma to hopefully create new treatment options with fewer side effects for patients.

**Methodology:**

CuO bio-NPs were produced by the addition of 15 mL of extract dropwise to 80 mL of a 5 mM Cu (OAc)_2_ aqueous solution, which was then refluxed for 2 h at 100 °C. The mixture gradually became darker brown in color as a result of the heating procedure. The production of CuO NPs and the hydrogen-donating activity of antioxidant phenols within the plant are signaled by surface plasmon resonance excitation, which is the cause of this. In the cell culture, LS174t colon cancer cells were treated with OB extract, CuNPs, and OB-coated CuNPs with and without different radiation levels in order to assess cell viability, through the MTT assay, and the pro-apoptotic BAX and anti-apoptotic BCL2 expressions, through qPCR assay.

**Results:**

The results demonstrate a decrease in cell viability and the expression of *BCL2* and an increase in the expression of *BAX* especially when treated with OB-coated CuNPs and even furthermore when paired with radiation therapy.

**Conclusions:**

After doing the clinical trial studies, the recent nanoparticles can be used for the treatment of Colorectal carcinoma.

## Highlights

• Effect of Ocimum bassilicum extract, Cu NPs, and Ocimum bassilicum -coated CuNPs on LS174t colon cancer cells.
• Cytotoxic effects of Ocimum bassilicum -coated CuNPs against LS174t cells.
• Intensifier effects of radiotherapy at these survival cells.

## 1 Introduction

Cancer originates from the conversion of healthy cells into neoplastic cells through a complex series of stages, encompassing the progression from a precancerous anomaly to a state of malignancy. colon cancer is the third most deadly diagnosed tumor globally, with Almost 2 million new cases. More than half of all colorectal cancers are attributed to the interplay between factors of genetic predispositions and various environmental stimuli, including physical agents such as ultraviolet radiation, and chemical substances like tobacco and alcohol, as well as biological factors such as viruses and bacteria. Common ways of treating colon cancer, depending on the doctor's diagnosis and the stage of the disease, include surgery, radiation therapy, and chemotherapy. Colon cancer consists of stages 0–4, in which treatment from stages 0–3 is surgical, and in stage 4, chemotherapy, radiation therapy, and surgery are used together [1]. Chemotherapy is a form of cancer treatment usually administered as an adjuvant or neoadjuvant to treat cancer with a combination of drugs. Its purpose is to increase the patient's life expectancy, prevent the spread of cancer, and eliminate some of the symptoms of cancer, which is carried out as palliative treatment. Chemotherapy drugs are usually injected non-specifically and spread throughout the body [2]. These drugs exert cytotoxic effects as they penetrate the cellular membrane, impeding the process of cell proliferation and growth in cancerous cells. This inhibition occurs through the interference with mitosis and cellular division, as well as the disruption of DNA structure and function, ultimately hindering DNA repair and regeneration [3]. It can also have negative side effects on healthy cells and tissues in the body, such as bone marrow, the gastrointestinal tract, and hair follicles, leading to hair loss, bone marrow failure, immune suppression, skin problems, nail problems, and mucositis. X-ray, gamma ray, and high-energy particles are extensively used in the practice of radiation therapy, a medical procedure implemented to treat various cancers. Ionizing radiation therapy is used to treat cancer. Ionizing radiation reacts with DNA either directly or indirectly. Direct interaction occurs through the direct interaction of charged particles with the cell, but indirect interaction occurs through the interaction of chargeless particles, including photons with H_2_O molecules and the formation of free radicals. In this study, indirect interaction is considered [4]. Radiation therapy is one of the most common methods of cancer treatment, which stops the growth of cancer cells and causes them to die by transmitting the radiation by creating gaps in the DNA inside cancer cells.

The purpose of radiation therapy is to deliver the highest dose to the tumor to increase the efficiency of treatment and to receive the lowest dose from the normal tissue to protect it [5]. However, the main disadvantages of radiation therapy include the radiation resistance of cancer cells due to DNA repair in tumors, the relatively low ability of radiotherapy to deliver therapeutic doses specifically to tumors, and the high probability of damage to healthy tissues. Therefore, radiation therapy is limited by the loss of healthy tissue because no differentiation is made between malignancies and healthy tissue. So, damage to healthy tissue along with tumor tissue is inevitable [[6], [7], [8]].

To overcome these limitations, new radiation sensitization strategies are the main challenge to improve the outcome of radiotherapy. Radiation sensitizers by increasing radiation efficiency cause the patient to receive a lower radiation dose and the surrounding healthy tissue is less damaged [9]. To reduce the toxicity of the surrounding healthy tissue and thus the toxicity of the surrounding healthy tissue. Recently, a lot of studies have been done to improve the effect of radiation therapy in fields such as advanced nanomaterials, nanobiotechnology, and nanomedicine [10]. Nanomaterials have good biocompatibility, inherent radiosensitizing activity, increased permeability of tumor tissue, etc [11]. The use of nanomedicine can greatly decrease the common limitations of chemotherapy drugs such as solubility, stability, and targeting. This makes nanomaterials a crucial part of alternative chemotherapy treatment methods and the recent focus of researchers on this subject can be a testament to their immense potential [12]. Furthermore, to replace the common chemotherapy medicine with alternative treatment options that have fewer side effects, researchers have been looking into traditional medicine [13]. traditional medicine, however, usually suffers from a few major disadvantages that limit its use including targeting and permeability but the incorporation of nanomaterial into them can greatly change these shortcomings [14].

Nanoparticles with higher density and atomic number, due to their larger absorption and scattering cross-sections, produce secondary electrons such as Auger, Compton, and photoelectrons that amplify the effect caused by radiation, so they are used as radiation sensitizers [15]. Also, nanosensitizers increase the sensitivity of tumors to radiation by producing reactive oxygen species. Recently, nanoparticle radiosensitizers, for example, gold, gadolinium, hafnium, silver, platinum, etc. have been extensively investigated as radiosensitizers [16]. In this work, we study the anticancer effects of Ocimum basillicum (OB)-coated copper (Cu) nanoparticles (CuNPs) on the colon carcinoma cell line LS174t with radiation and without different radiation levels and evaluate the expression of pro-and anti-apoptotic genes.

## 2 Experimental

### 2.1 Materials

Cu(CH_3_COO)_2_. H_2_O (100 % purity) was purchased from Merck Co. Without any purification, all other analytical grade compounds were employed.

### 2.2 Instrument analysis

The Bruker spectrophotometer was employed for Fourier transform infrared (FT-IR) spectra analysis at room temperature in the 400–4000 cm-1 range. High-resolution scanning electron microscopy (LEO 1455VP) was used for surface morphology assessment of synthesized nanoparticles. The produced nanoparticle compounds were evaluated using energy-dispersive X-ray spectroscopy (EDX). A Bruker D8 Advance diffractometer recorded the compounds' X-ray diffraction (XRD) pattern.

### 2.3 Preparation of CuO bio-nanoparticles (CuO-bio-NPs)

In the initial step, Ocimum basillicum leaves were gathered from Tabriz, Iran and subsequently, they were air-dried in a dark environment. The dry leaves were powdered and heated at 40 °C for 2 h 1800 ml of distilled water (weight to volume ratio 6.1) was added to 300 g of the dried powder and after keeping it in the room for 24 h, it was filtered with Whatman 2 paper. After filtering, this extract was evaporated in a vacuum distillation apparatus for an hour at 80 °C. To remove any bacterial contamination, the finished extract was sonicated and passed through 0.2 μm filters.

CuO bio-NPs were produced by the addition of 15 ml of extract dropwise to 80 ml of a 5 mM Cu (OAc)_2_ aqueous solution, which was then refluxed for 2 h at 100 °C. The mixture gradually took on a dark brown tint as a result of the heating process. This is because the plant's antioxidant phenols donate hydrogen through their surface plasmon resonance excitation, which signals the creation of CuO NPs. After that, the precipitate was cleaned three times using ethanol and chloroform, respectively, and allowed to air dry for 48 h at room temperature [17].

### 2.4 Radiation

**Image1:**
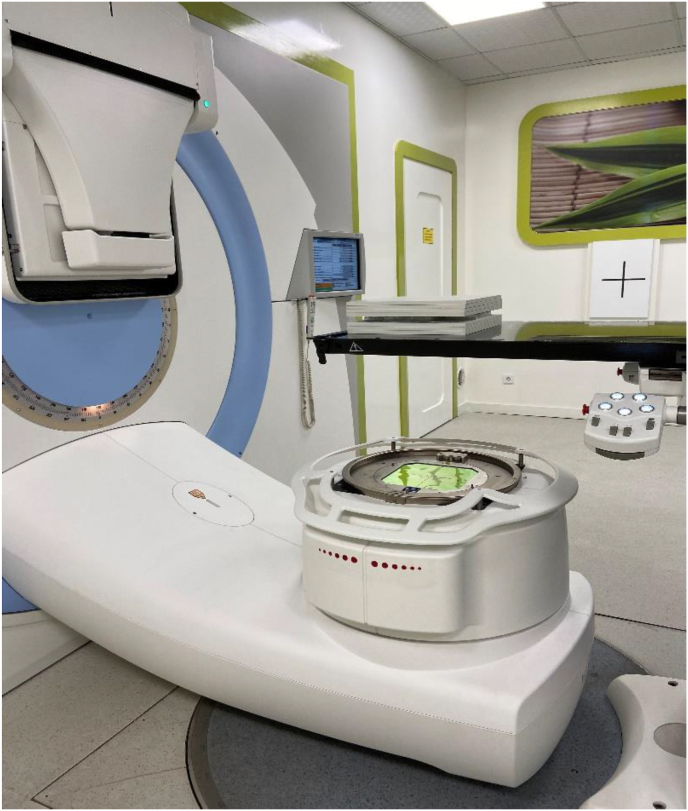
Radiation setup.

Using the Elekta Linac from Sweden, X-ray irradiation with high-energy levels at 6 and 15 MV was performed on cells. This procedure took place within the radio oncology department of Tabriz University of Medical Sciences, whereby a radiation dose of 2Gy was administered at a Source-to-Surface Distance (SSD) of 100 cm (Image1I).

### 2.5 Anticancer analysis

The Pasteur Institute of Iran supplied the HCT116 normal colon cell line, along with the LS174t and COLO205 human colon cancer cell lines, for research purposes. To improve adhesion, these cells were cultured in RMPI1640 medium on gelatin-coated plates, with the addition of specific components like fetal bovine serum, antibiotics, 1 % l-glutamine, and 1 % non-essential amino acids. Finally, the cells were kept in a 37 °C incubator with 5 % CO_2_.

#### 2.5.1 Cell viability

Cell vitality was determined using the methyl thiazol tetrazolium (MTT) assay with the MTT kit from the Cytotoxicity Test Kit (Arsamsysbio.com, Iran). After 24 h, 3000 cells were seeded in 96-well plates and treated with different concentrations (500, 1000, 2000, and 4000 μg/ml) of OB-coated CuNps. At the 24, 48, and 72-h time points of incubation, the wells were exposed to 10 % MTT (3-(4,5-dimethylthiazol-2-yl)-2,5-diphenyltetrazolium bromide). After an additional 4-h incubation period, the wells underwent drainage, followed by adding a formazan solubilizing solution, and subsequent measurement of absorbance at 560 nm.

#### 2.5.2 mRNA extraction

ZiAzole (ZiAViZ company, Iran) mRNA extraction kit was used to extract mRNA in this study. Cells were mixed with 1 ml of ZiAzole and left to stand before vortexing. After the cells were allowed to reach room temperature for 5 min, 200 μL of chloroform was added and mixed vigorously for 15 s. After mixing with ice, the resultant mixture underwent centrifugation (12,000 rpm for 10 min) and the supernatant solution was combined with is-propanol (1 mL) in a sterile microtube. The mixture was gently shaken and incubated on ice for 15 min, then centrifuged to get phase separation (12,000 rpm for 15 min). The top layer was replaced with 1 mL of 75 % ethanol, which was then gently shaken. The supernatant solution was removed and the residual substances after being centrifuged for 10 min at 12,000 rpm were dried at room temperature. Following resuspension of the mixture in 50–100 μL of sterile water, was stored at room temperature for 30 min, and then was placed in a freezer.

#### 2.5.3 Reverse transcription PCR

The cDNA Synthesis Kit (Yekta Tajhiz, Iran) was used in this assay to obtain cDNA from mRNA. 1 μL of oligo(dT) primer, 5 μL of sterile water, and 5 μL of mRNA (decided upon through nanodrop test) were mixed. This mixture was heated at 70 °C for 5 min before being placed on the ice-cold rack. For every sample, 6.5 μL of PCR master mix (made in accordance with kit instructions) was added. In the final RT-PCR process, the sample was heated at 42 °C for an hour, followed by a 5-min treatment at 70 °C.

#### 2.5.4 qPCR assay

The expression of the pro-apoptotic BAX and anti-apoptotic BCL2 genes was evaluated using a qPCR technique. Yekta Tajhiz qPCR kit was utilized in this assay and instructions given by the kit were followed throughout the assay. In this assay, LS174 t-cells were treated with 0.5, 1, 2, and 4 μg/ml concentrations of OB extract, CuNps, and OB-coated CuNPs with and without different doses of radiation. The expression of the beta-actin housekeeping gene was evaluated as a reference gene. Table I shows the primer sequences.

**Table 1:** qPCR of Primer sequences.

Gene	Reverse	Forward
BAX	5′– GCCACGTGGGCGGTCCCAAAGT – 3′	5′- GGCCCACCAGCTCTGAGCAGA- 3′
Bata actin	5’ – CTAGAAGCATTTGCGGTGGA – 3′	5′- GGAGTCCTGTGGCATCCACG- 3′
Bcl-2	5′- AGGCACCCAGGGTGATGCAA-3′	5′- GTGGAGGAGCTCTTCAGGGA-3′

## 3 Results and discussion

### 3.1 Characterization of preparated materials

#### 3.1.1 FT-IR study and XRD analysis

**Fig. 1:**
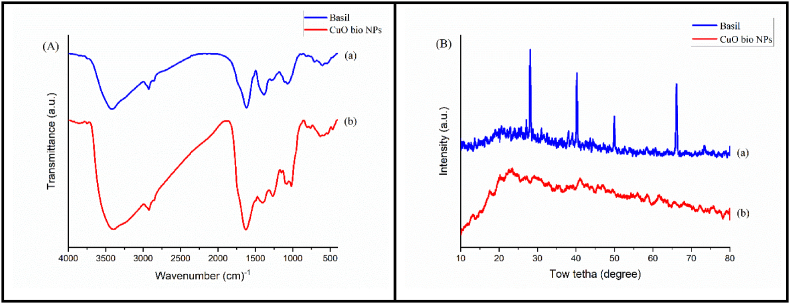
(A) FT-IR spectrum of Basil extract (a), CuO bio nanoparticles (b) and (B) XRD spectrum of Basil extract (a), CuO bio nanoparticles (b).

Fig. 1 (A) depicts the FT-IR spectra of the involvement of biological entities in the extract in copper salt reduction and nanoparticle capping. The stretching bands of C–*O*–H and C–O are visible in the basil extract's spectrum at 1069 cm-1 and 1282 cm-1, respectively. At 1384 cm-1, all of the active methylene flexing is seen as a sequence of sharp, intense bands. What distinguishes the carbonyl group is the strong signal in the 1618 cm-1 region. The methyl group's C–H stretch is shown by the peak at 2925 cm-1, and the O–H stretch is represented by a high, broad peak at 3419 cm-1 (Fig. 1A(a)). CuO nano-particles spectra (Fig. 1B) showed that adsorption bands at 3393 and 2923 cm-1 correlated to the O–H and C–H stretching vibration and adsorption bands at 3393 and 2923 cm-1 related to the O–H and C–H bending. In addition, the characteristic CuO peak was found at 634 cm^−1^ [15]. These outcomes demonstrate the decreasing and limiting effects of organic matter in basil (Fig. 1A(b)). [18].

Fig. 1B illustrates the X-ray diffraction (XRD) of the CuO bionanoparticles production. The XRD diffractogram of the raw basil extract (Fig. 1B(a)) exhibited four prominent diffraction peaks at 2θ angles of 28.1°, 40.22°, 49.94°, and 66.08°. These peaks may be to be linked to the extract's crystal structure. In Fig. 1B(b), the XRD spectrum of the CuO bionanoparticles reveals the absence of diffraction peaks, affirming the amorphous nature of these green synthesized nanoparticles. The 2θ value of 24° broadband may be caused by various biomolecules in the plant extract that serve as capping agents.

#### 3.1.2 SEM study

**Fig. 2:**
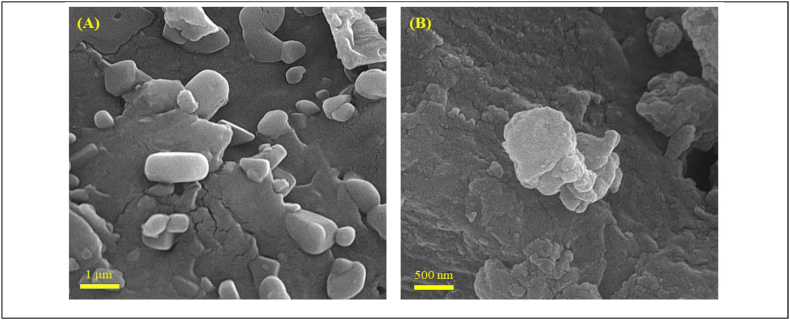
SEM images of (A) Basil extract, (B)CuO bio nanoparticles.

SEM (micrographs with 1 mm and 500 magnification) was utilized to examine the surface morphology and structure of the CuO bio nanoparticles and basil extract (Fig. 2). As shown in Fig. 2A, the basil extract displays a smooth surface morphology, while the CuO bio nanoparticles exhibit a coarse texture, indicating the existence of CuO nanoparticles on the surface of the plant extract (Fig. 2B).

#### 3.1.3 EDS analysis

**Fig. 3:**
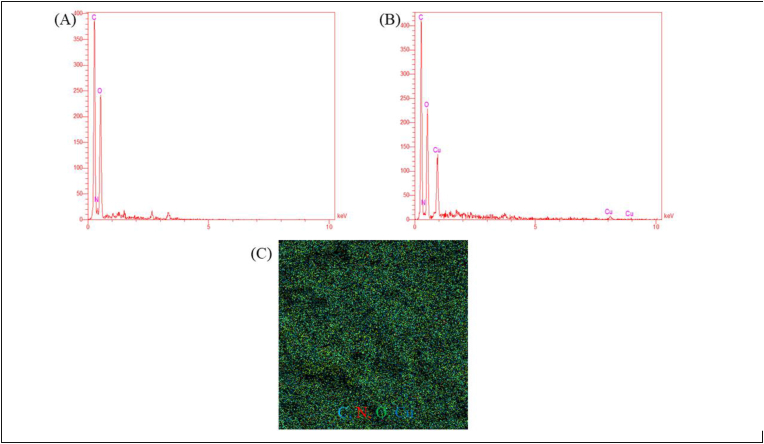
EDS spectrum of Basil extract (A), CuO bio nanoparticles (B), and Mapping image of CuO bio nanoparticles (C).

The elemental composition of the substances produced was examined through the utilization of the EDS technique. (Fig. 3). CuO bio-NPs' existence in the EDS spectrum in comparison with to basil extract (Fig. 3A) demonstrated that these nanoparticles had successfully formed (Fig. 3B). Also, the mapping image of CuO bio nanoparticles shows that different amounts of oxygen, carbon, copper, and nitrogen atoms are present (Fig. 3C).

### 3.2 Anti-colon cancer effects of nanoparticles

Cell viability was performed to assess the viability of our cells after treatment with no radiation, 2Gy and 6 MV of radiation, and 2Gy and 15 MV of radiation. All these steps were repeated for 24h, 48h, and 72 h. In each step, the cells were treated with OB extract alone, OB-free nanoparticles, and OB-coated nanoparticles. Table 2 shows the IC50 values.

**Table 2:** IC50 as presented for cell viability (MTT) test.

	OB	NPs	NPs + OB
	24 h of treatment (μg/ml)		
Without Radiotherapy	1523	2219	1498
With 2Gy and 6 MV of Radiotherapy	1568	2006	1368
With 2Gy and 15 MV of Radiotherapy	1773	1815	1223
	48 h of treatment (μg/ml)		
Without Radiotherapy	2531	2753	1441
With 2Gy and 6 MV of Radiotherapy	1519	1941	1263
With 2Gy and 15 MV of Radiotherapy	1659	1868	1261
	72 h of treatment (μg/ml)		
Without Radiotherapy	2250	2276	1190
With 2Gy and 6 MV of Radiotherapy	1611	1956	1357
With 2Gy and 15 MV of Radiotherapy	1662	2147	1325

**Fig. 4:**
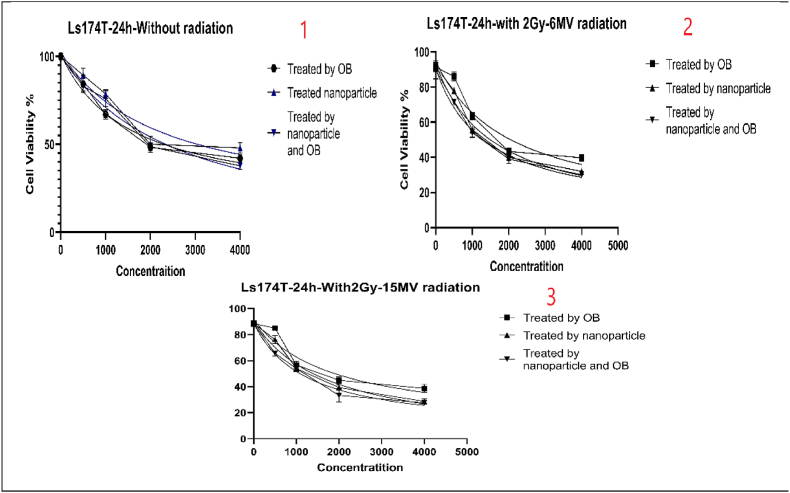
Cell viability results after the first day of *treatment in multiple settings.*

**Fig. 5:**
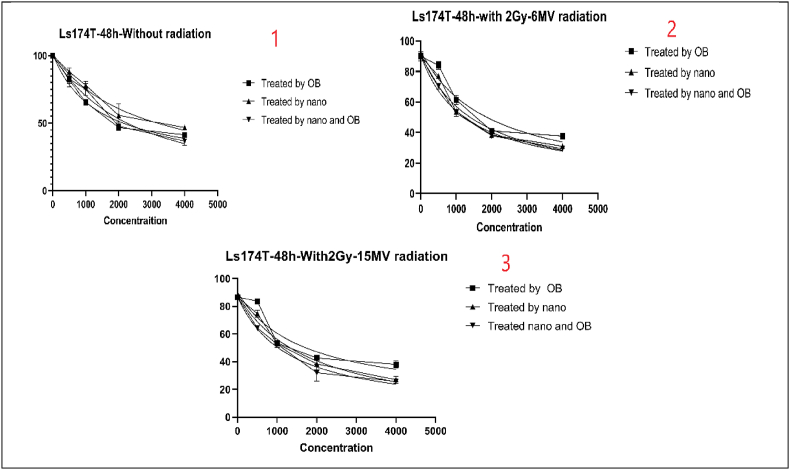
Cell viability results after the second day of treatment in multiple settings.

**Fig. 6:**
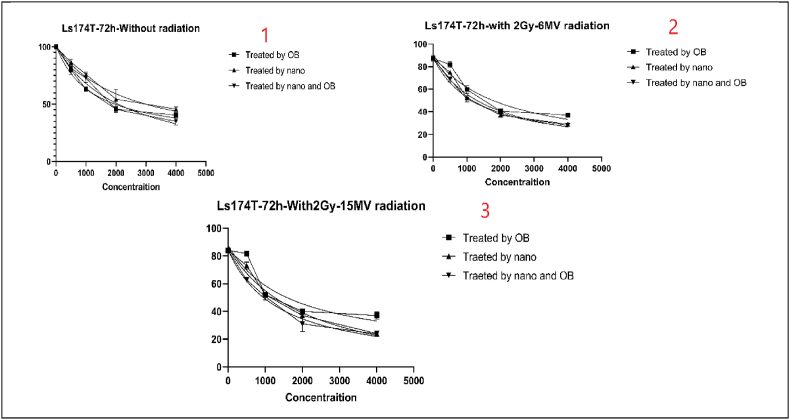
Cell viability results after the third day of treatment in multiple settings.

The cell viability graphs after the first, second, and third day of treatment are presented in Fig. 4, Fig. 5, Fig. 6 respectively.

These results can be interpreted in various manners. First of all, there is an obvious decrease in cell viability the longer the treatment is applied. Meaning that there is a negative correlation between the duration of treatment and cell viability. secondly, OB-coated NPs present better cytotoxicity and can deal greater damage to cancer cells than OB extract alone or just NPs. This means that OB-coated NPs have a greater potential to fight cancer cells. Third and finally, the outcomes show that the combination of treatment, with OB extract and OB-coated NPs, can further improve the cytotoxic effects of the treatment. This effect is greater the more powerful the radiation gets. Overall, the best results were achieved when the cells were treated with OB-coated NPs along with 2Gy and 6 MV of radiation after 24, 48, and 72 h, especially at the 72-h mark.

**Fig. 7:**
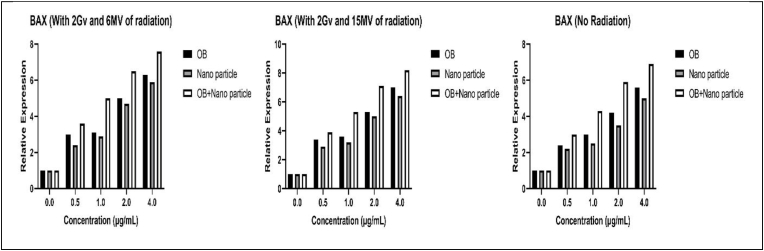
Changes in the expression of the BAX gene with and without different radiation levels and after various treatment methods.

**Fig. 8:**
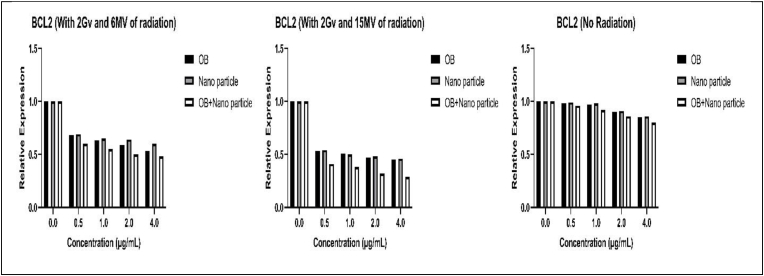
Changes in the expression of the BCL2 gene with and without different radiation levels and after various treatment methods.

The expression of BAX and BCL2 was evaluated using a qPCR assay following treatments with varying dosages of OB extract, CuNPs, and OB-coated CuNPs, both with and without varying radiation levels. Fig. 7, Fig. 8 demonstrate the changes in the expression of pro-apoptotic BAX and anti-apoptotic BCL2 respectively. Table 3, Table 4 include p-values for changes in the expression of these genes (see Fig. 9).

**Table 3:** P-Values for the qPCR result of the BAX gene (P-Values <0.05).

	P-Values			
	0.5	1	2	4
BAX _No radiation				
OB extract	0.0101*	0.005**	0.0019**	0.0009***
CuNPs	0.0136*	0.0088**	0.0032**	0.0012**
OB-coated CuNPs	0.005**	0.0018**	0.0008***	0.0006***
BAX_With 2Gy and 6 MV of radiation				
OB extract	0.005**	0.0045**	0.0012**	0.0007***
CuNPs	0.0101*	0.0055**	0.0015**	0.0015**
OB-coated CuNPs	0.0029**	0.0012**	0.0007***	0.0005 ***
BAX_With 2Gy and 15 MV of radiation				
OB extract	0.0035**	0.0029**	0.0009***	0.0006***
CuNPs	0.005**	0.0041**	0.0012**	0.0007***
OB-coated CuNPs	0.0024**	0.0011**	0.0005***	0.0004***

**Table 4:** P-Values for the qPCR result of the BCL2 gene (P-Values <0.05).

	P-Values			
	0.5	1	2	4
BCL2_No radiation				
OB extract	ns	ns	ns	ns
CuNPs	ns	ns	ns	ns
OB-coated CuNPs	ns	ns	ns	ns
BCL2_With 2Gy and 6 MV of radiation				
OB extract	ns	ns	ns	0.0428*
CuNPs	ns	ns	ns	ns
OB-coated CuNPs	ns	0.0464*	0.0381*	0.0345*
BCL2_With 2Gy and 15 MV of radiation				
OB extract	0.0035**	0.0029**	0.0009***	0.0006***
CuNPs	0.005**	0.0041**	0.0012**	0.0007***
OB-coated CuNPs	0.0024**	0.0011**	0.0005***	0.0004***

As the results demonstrate, the expression of pro-apoptotic *BAX* increases after treatment with our components. The increase in expression is most evident when the cells are treated with OB-coated CuNPSs followed by OB extract and then the CuNPs alone. Furthermore, the addition of radiation especially at higher levels further increases the expression of this gene. As for the anti-apoptotic *BCL2,* we observe the exact opposite during the same process and in the same manner. We see a decrease in its expression. As a whole, we can conclude that the apoptotic activity of the cells may be positively affected by our molecules, especially OB-coated CuNPs, and this result can be further improved by adding radiation to this equation.

As mentioned previously, colon cancer is raging over the modern world, and common methods of treatment, most notably chemotherapy, are cumbersome processes for the patients physically, mentally, and financially. Using traditional medicine elements can help with lowering the side effects. Using nanoparticles, however, can increase the efficiency of the treatment while lowering the dosage needed for the treatment thus lowering the side effects even more. This study aimed to compare the Cu NPs, OB, and OB-coated Cu NPs in fighting colon cancer cells with and without the help of radiation therapy.

Our results presented that all methods of treatment used in this study can effectively lower the viability of colon cancer cells and can hypothetically increase apoptotic activity by increasing the expression of pro-apoptotic *BAX* and decreasing the expression of anti-apoptotic *BCL2*. The best results arose from OB-coated NPs, OB, and NPs respectively. These results also demonstrated that treatment accompanied by radiation has considerably better results and more toxicity compared with treatment alone, and the higher the radiation becomes the lower the cell viability drops.

The use of nanoparticles carrying basil compounds for anticancer purposes is an ongoing area of research in the field of nanomedicine. Basil, especially its active compounds such as flavonoids and essential oils, has been researched for its potential anticancer properties because of its antioxidant, anti-inflammatory, and anti-proliferative effects. Nanoparticles can improve the delivery of basil compounds to cancer cells, increasing bioavailability and targeting specific cellular pathways involved in cancer progression. This targeted approach has the potential to reduce the side effects associated with conventional cancer treatments while also increasing the efficacy of the treatment [19]. Manikandan et al. used silver nanoparticles with leaf extract to investigate anticancer activity on the A549 human lung cancer cell lines. In this study, using fluorescent staining techniques and flow cytometry analysis, they showed an increase in cytotoxic activity and apoptosis, which was characterized by G0/G1 cell cycle arrest [20]. Also, the findings show that basil extracts, due to their ability to induce cell death and disrupt the progress of the cell cycle in cancer cells, play an important role in the process of apoptosis.

Recently the study showed that basil leaf extract induces apoptosis in pancreatic cancer (PC) cell lines (AsPC-1, MiaPaCa, and Capan-1) in vitro. this extract prevents the proliferation, migration, and invasion of cancer cells. In addition to inducting apoptosis, the study also found that the genes responsible for promoting survival and resistance to chemotherapy were reduced [21]. Elansary and Mahmoud conducted a separate investigation on various basil extracts to examine their effectiveness in inhibiting the growth of human cancer cells. All basil extracts caused apoptosis and cell cycle progression, resulting in anti-proliferation and cell death. As a result, all basil extracts caused apoptosis and cell cycle progression, whose properties are probably related to bioactive compounds such as rosmarinic or capharic acids [22]. A study done by Gulshan Dhillon et al. investigated the cytotoxic effects of Ocimum basilicum leaf extract-mediated Fe 3 O4 NPs on MDA-MB-231 breast cancer cells. This study concluded that OB-Fe3O4 NPs have great cytotoxic potential against breast cancer cells [23]. In a research in 2019, silver-montmorillonite nanocomposites constructed using Ocimum Basilicum were put to test against *E. coli*, *S. aureus*, and HEP G2 liver cancer cells. These nanocomposites induced an acceptable amount of anti-microbial and cytotoxic effects on these bacteria and cancer cells [24].

Asha Monica A et al. also tested the anti-microbial and anti-cancer effects of silver nanoparticles mediated by Ocimum basilicum aqueous leaf extract on *Escherichia coli and Klebsiella pneumonia* and MCF-7 breast cancer cells. The outcomes of this work also support our findings like the studies mentioned before [25]. Combined results of our study and findings from limited studies on these subjects that are available conclude that the combination of OB extract and NPs can be a lethal weapon against cancer cells. OB extract alone has potent cytotoxic effects on colon cancer cells [26]. Using NPs to deliver this extract improves the specificity and efficiency of the treatment and adding radiation therapy to the mix only further improves the positive results of this treatment on colon cancer lines [27]. Further research is required to additionally study the cytotoxic and anti-cancer influences of OB-coated Cu NPs on colon cancer and other cancers to provide a better future for the treatment of cancer patients more efficiently.

## 4 Conclusion

Today, the use of plant extracts (due to their antioxidant properties) for the green synthesis of nanoparticles has received much attention. The CuO-bio-NPs used in this work are usually prepared by continuously stirring 15 mL of Ocimum basilica leaves with 80 mL of Cu(OAc)_2_ aqueous solution (5 mM) for 2 h at 100 °C. Various analyses such as FT-IR, XRD, SEM, EDS, and UV were used to confirm the synthesis of these nanoparticles. In different sample concentrations, the MTT method was used to analyze the cytotoxicity of colon cancer cell lines. It was found that there was a dose-dependent decrease in the percentage of viable cells. This shows why copper bionanoparticles can be a suitable option for a nano-formulated drug against various forms of colon cancer. We will be happy to do more research on this topic to explore a new angle of cancer research. Green synthesis of other nanoparticles, including silver nanoparticles, iron nanoparticles, zinc oxide, etc., using extracts with more antioxidant properties, can be very effective in the field of cancer treatment.

## Ethics approval and consent to participate

This study was approved by the Ethical Committee of Tabriz University of Medical Sciences (IR.TBZMED.VCR.REC.1400.199) and (No.66937). Also, this study was funded by the Molecular Medicine Research Center of 10.13039/501100004366Tabriz University of Medical Sciences.

## Consent for publication

Not applicable.

## Availability of data and materials

Not applicable.

## Competing interests declaration

Not applicable.

## Funding

This research received no external funding

## CRediT authorship contribution statement

**Farshad Seyed Nejad:** Supervision, Data curation. **Mostafa Alizade-Harakiyan:** Writing – review & editing, Writing – original draft, Investigation, Conceptualization. **Mehdi Haghi:** Supervision, Investigation, Data curation. **Rokhsareh Ebrahimi:** Writing – review & editing, Writing – original draft, Validation, Investigation. **Mohammad Mahdi Zangeneh:** Writing – original draft, Investigation, Conceptualization. **Alireza Farajollahi:** Visualization, Validation, Supervision, Resources. **Roghayeh Fathi:** Writing – original draft, Methodology, Formal analysis. **Reza Mohammadi:** Resources. **Samira Samadi Miandoab:** Resources. **Mohammad Heydarnezhad Asl:** Writing – original draft, Software, Resources. **Baharak Divband:** Resources. **Amin Ahmadi:** Resources.

## Declaration of competing interest

Not applicable.

## Data Availability

Availability of data and materials: Not applicable.
